# Subarachnoid hemorrhage triggered by spinal cord vascular malformation in a pediatric patient: case report and review of literature

**DOI:** 10.3389/fsurg.2024.1487979

**Published:** 2025-01-06

**Authors:** Ye Zhang, Liaoyuan Zheng, Yongwei Huang, Zongping Li, Jing Feng

**Affiliations:** ^1^ Department of Neurosurgery, Mianyang Central Hospital, School of Medicine, University of Electronic Science and Technology of China, Mianyang, Sichuan, China; ^2^ Department of Breast Surgery, Mianyang Central Hospital, School of Medicine, University of Electronic Science and Technology of China, Mianyang, Sichuan, China

**Keywords:** spinal cord vascular malformations, subarachnoid hemorrhage, digital subtraction angiography, microsurgical, pediatrics, case report

## Abstract

**Background:**

Spinal cord vascular malformations (SCVMs) in children are relatively rare and present unique challenges due to their distinct physiological characteristics. These malformations often manifest with nonspecific clinical symptoms, increasing the likelihood of misdiagnosis. The treatment of pediatric SCVMs requires a tailored approach, with the choice between microsurgical intervention and endovascular embolization depending on the specific type of malformation and individual patient factors.

**Case report:**

We report a case of a 6-year-old male who presented with a sudden onset of headache. Initial cranial imaging did not reveal any significant intracranial vascular malformations. However, thoracic spine magnetic resonance imaging (MRI) identified an abnormal signal, and digital subtraction angiography (DSA) confirmed the diagnosis of SCVMs. The patient underwent microsurgical treatment and was discharged in good health. Follow-up DSA confirmed the complete resolution of the vascular malformations.

**Conclusion:**

This case, along with a review of the literature, underscores the importance of thorough spinal evaluations in pediatric patients with spontaneous intracranial hemorrhage, especially when intracranial vascular malformations are not identified. A high index of suspicion for SCVMs is crucial. Early and accurate diagnosis, followed by appropriate treatment through microsurgical resection or endovascular embolization, can significantly improve therapeutic outcomes in children with SCVMs.

## Introduction

Spinal cord vascular malformations (SCVMs) are congenital developmental anomalies of the spinal cord vasculature. These abnormal vascular formations can lead to spinal cord dysfunction through mechanisms such as local mass effect, thrombosis, vascular steal, and hemorrhage (1). Emerging research indicates that SCVMs are a significant cause of misdiagnosis, potentially leading to disability and death (2). SCVMs can be classified into various types based on lesion location and vascular supply characteristics, including dural arteriovenous fistulas (Type I), glomerular arteriovenous malformations (Type II), juvenile arteriovenous malformations (Type III), and perimedullary arteriovenous fistulas (Type IV) (3). The overall incidence of SCVMs in children is low, with considerable variation in reported prevalence across studies, likely due to differences in diagnostic criteria and patient age distribution. The insidious onset and nonspecific clinical manifestations of SCVMs contribute to a high rate of misdiagnosis. According to research by Jablawi et al. (4), the interval from symptom onset to diagnosis is closely associated with patient prognosis, underscoring the importance of early recognition, diagnosis, and treatment of pediatric SCVMs.

## Case description

**Figure 1:**
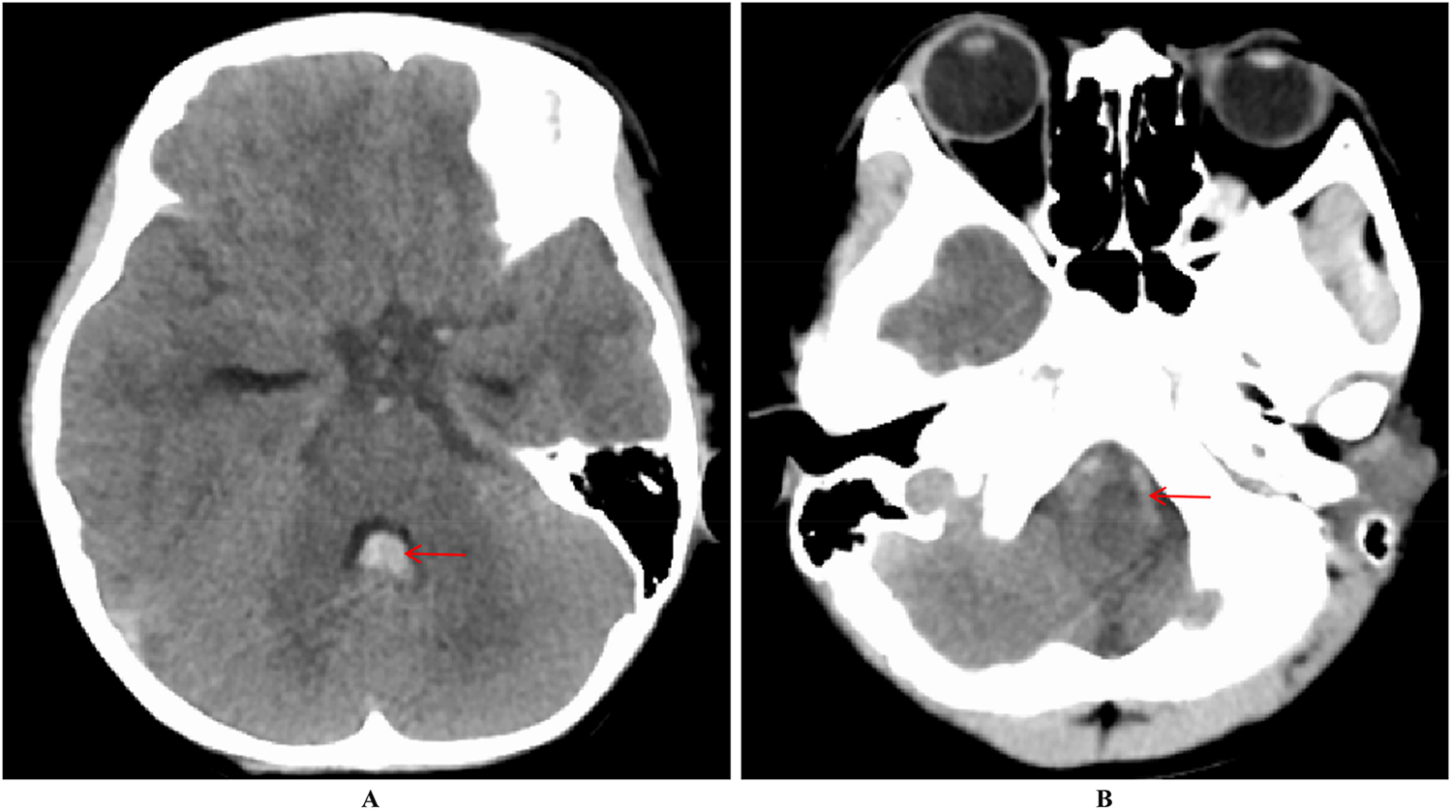
Increased density within the fourth ventricle **(A)** and a lamellar high-density shadow in the anterior part of the medulla oblongata **(B)** suggest hemorrhage.

**Figure 2:**
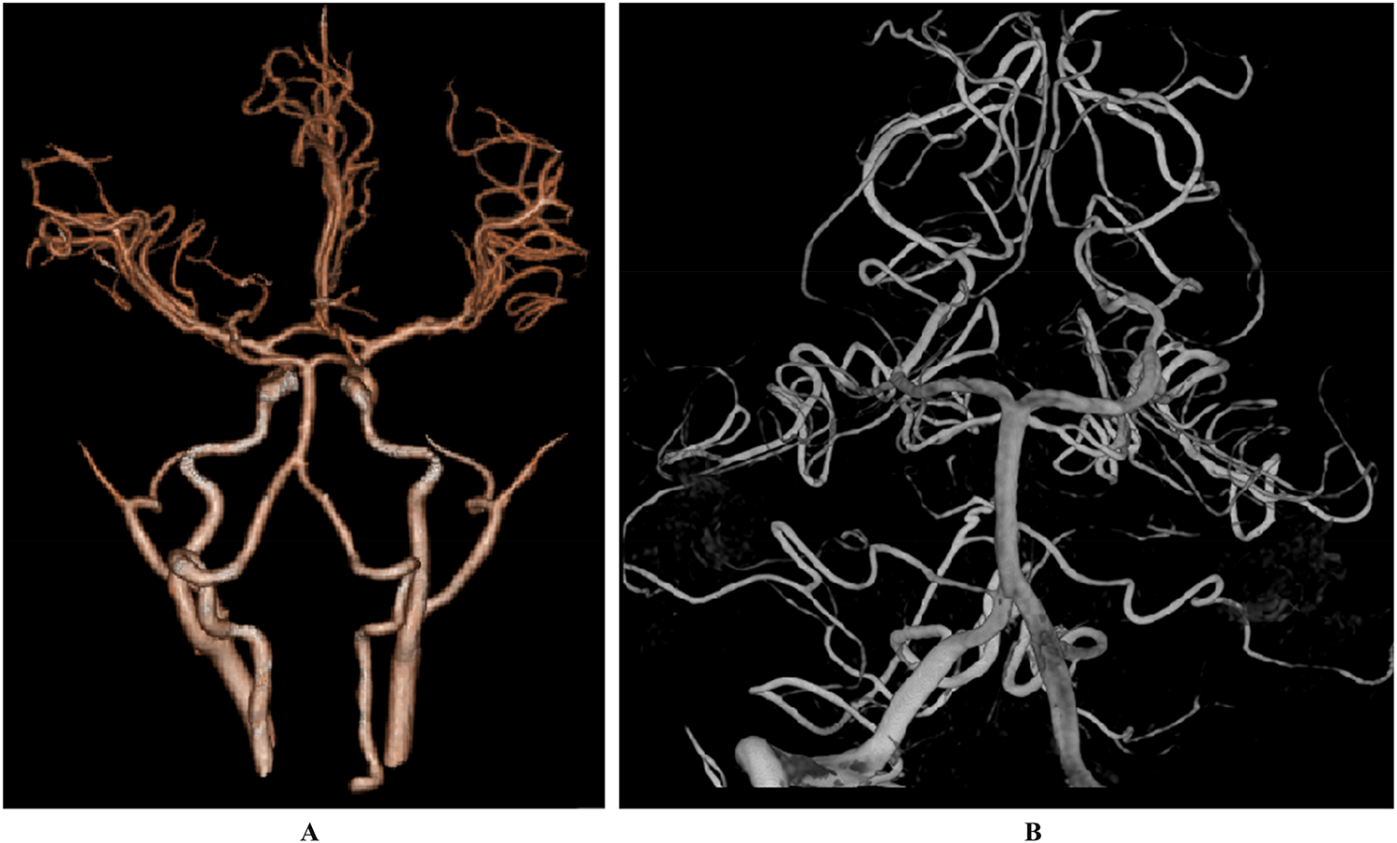
The results of CTA **(A)** and DSA **(B)** did not show any significant abnormalities.

**Figure 3:**
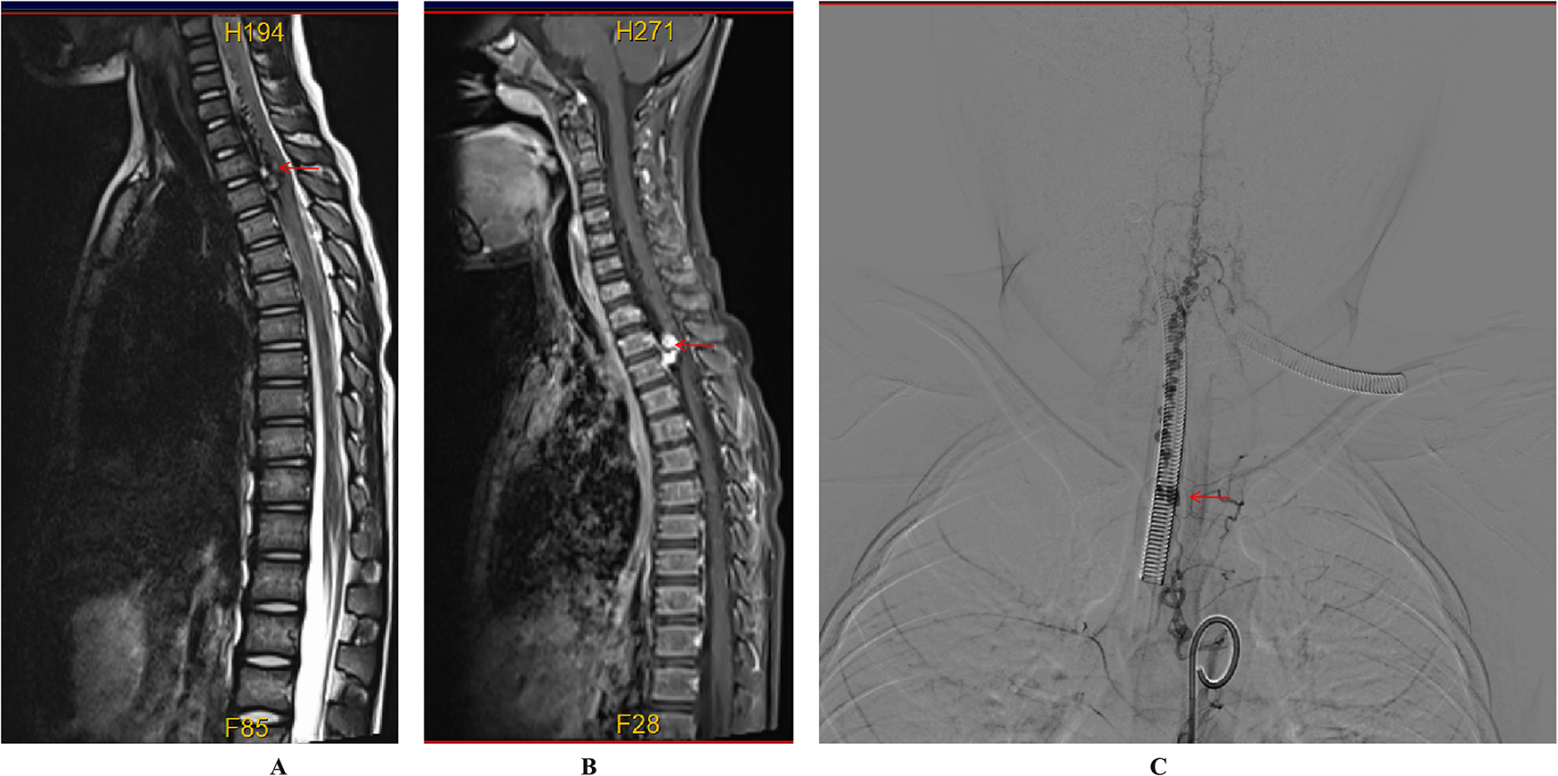
Preoperative imaging of the spinal cord vascular malformation. **(A)** T2-weighted MRI shows a slightly hypointense, tortuous signal beneath the extradural dura mater extending from the C4 to T3 levels. **(B)** Enhanced MRI reveals nodular enhancement, particularly at the T2–T3 level. **(C)** Digital subtraction angiography (DSA) demonstrates that the right intercostal artery at the level of the 6th to 7th rib drains into the intramedullary vein at the T2 level, with multiple tortuous and thickened vascular networks extending from the cervical segment to the T2 level of the spinal cord.

**Figure 4:**
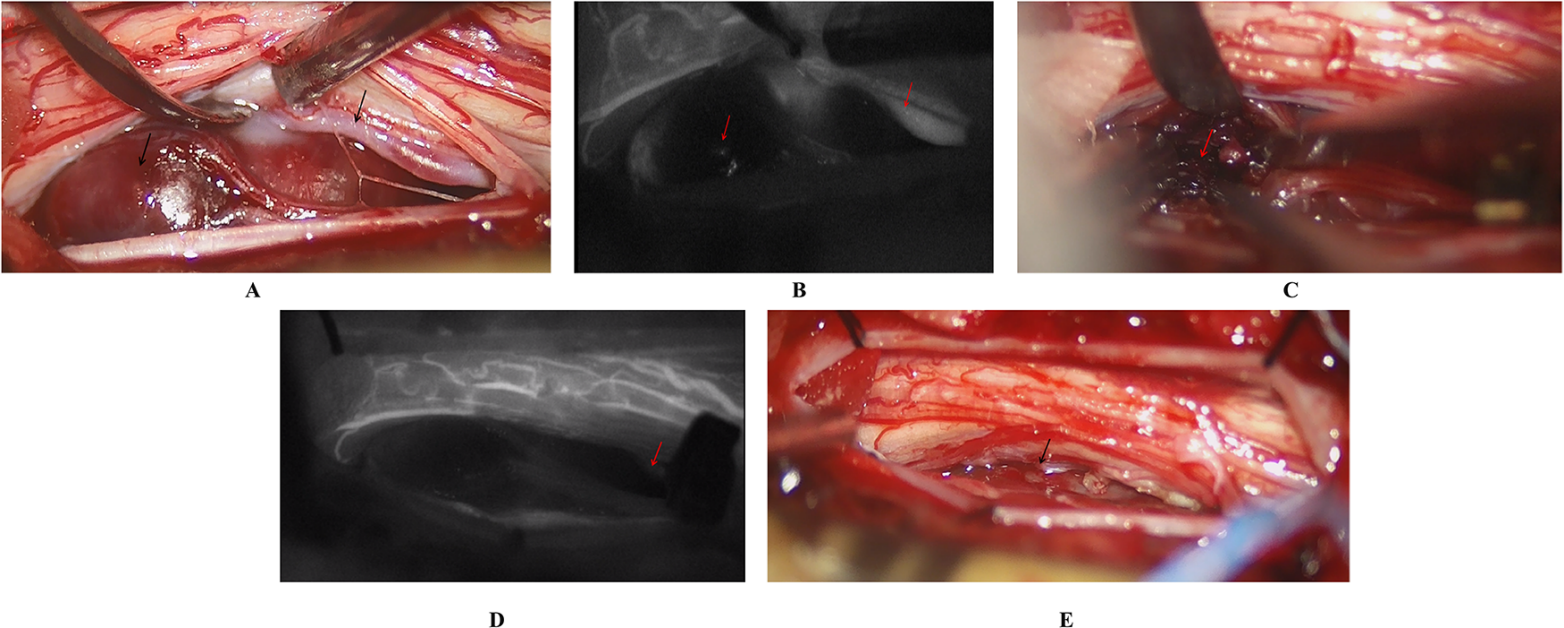
Intraoperative findings and management of the SCVMs. **(A)** Malformed blood vessels were observed on the ventral side of the spinal cord, accompanied by localized aneurysmal dilation. **(B)** Intraoperative fluorescence angiography revealed patency of the feeding artery, but no significant enhancement of the aneurysmal dilation. **(C)** The thrombus was identified within the aneurysmal dilation. **(D)** Temporary occlusion of the feeding artery was performed, and subsequent fluorescence angiography showed no enhancement of the malformed vessels. **(E)** Complete resection of the SCVM was achieved.

**Figure 5:**
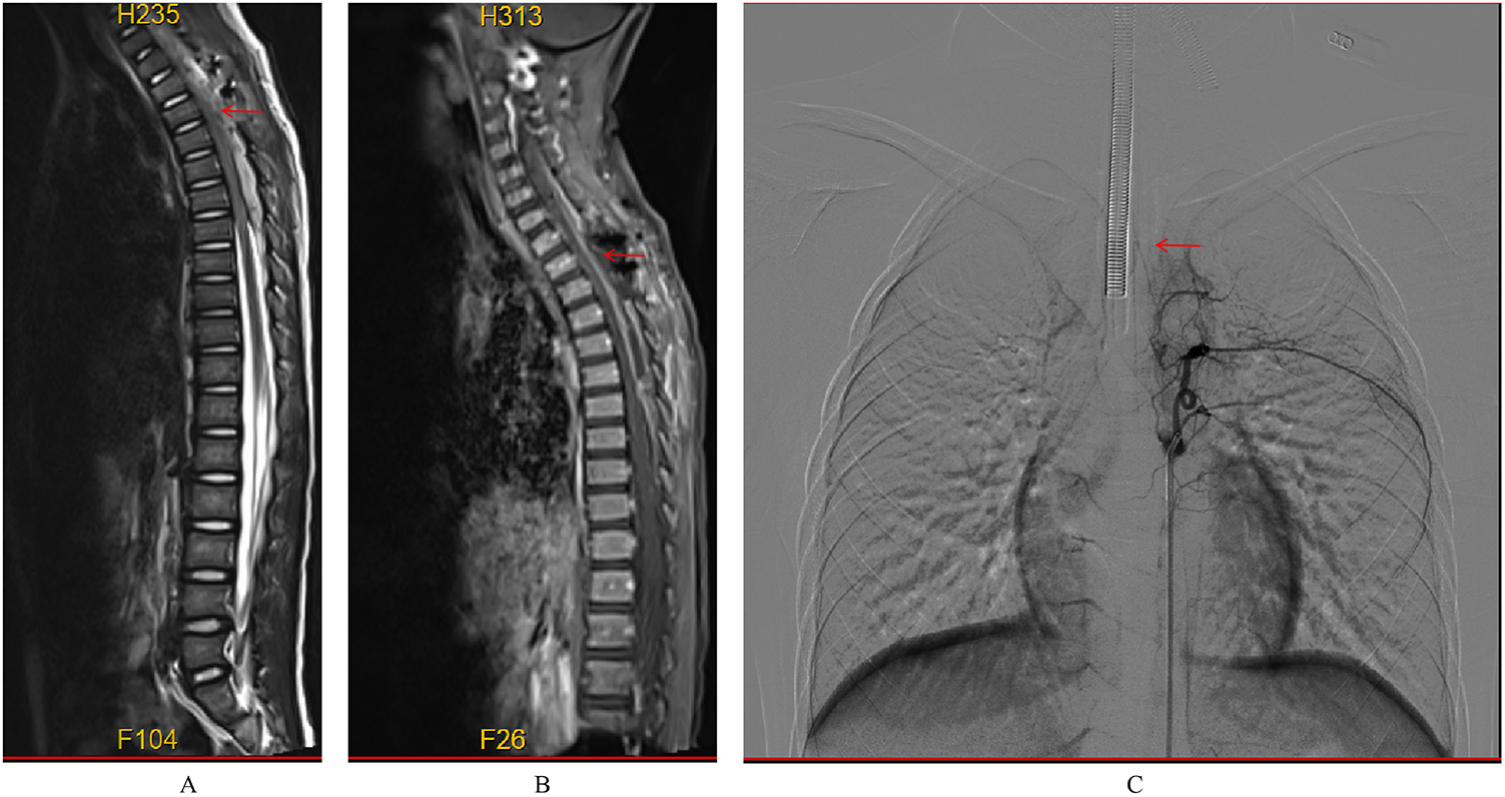
Postoperative MRI did not reveal any significant abnormal signals **(A,B)**; postoperative DSA showed no evidence of abnormal vasculature **(C)**.

A 6-year-old male presented with a sudden onset of headache, accompanied by nausea and vomiting, persisting for one day. On admission, cranial computed tomography (CT) revealed hemorrhage in the fourth ventricle and minor bleeding in the anterior portion of the medulla oblongata (Figures 1A,B). The physical examination was largely unremarkable, except for positive neck stiffness, with no other significant neurological signs. Following admission, cranial computed tomography angiography (CTA) and digital subtraction angiography (DSA) were performed, neither of which revealed significant intracranial vascular malformations (Figures 2A,B). Despite a week of conservative treatment, the patient continued to report neck discomfort. While subarachnoid hemorrhage was considered, based on our prior experience with spinal pathologies and a review of relevant literature, cervical myelopathy could not be ruled out. An initial cervical magnetic resonance imaging (MRI) was conducted, revealing abnormalities. Consequently, the MRI examination was extended to include the thoracic and lumbar regions. The MRI indicated an abnormal subdural extramedullary signal at the T3 vertebral level, characterized by slightly low signals on T1- and T2-weighted images. Enhanced imaging revealed significant, uneven enhancement (Figures 3A,B). Subsequent complete spinal angiography demonstrated drainage from the right T6-T7 intercostal artery to intramedullary veins, with a network of tortuous, thickened vessels extending from the T2 to the cervical segment of the spinal cord (Figure 3C). Given the findings, the child's intracranial hemorrhage was attributed to a spinal vascular malformation, and surgical intervention was deemed necessary. The malformation was resected using advanced microsurgical techniques, with continuous intraoperative somatosensory evoked potential monitoring and spinal vascular fluorescence angiography. Intraoperatively, the malformed vessels were located ventrally on the spinal cord, with localized aneurysmal dilation significantly compressing the surrounding spinal cord tissue (Figure 4A). Intraoperative fluorescence angiography revealed patency in the feeding artery, but the aneurysmal dilation showed no significant enhancement, suggesting thrombus formation. This was confirmed intraoperatively, as the aneurysmal dilation was filled with thrombus (Figures 4B,C). The feeding artery was temporarily occluded, and subsequent fluorescence angiography showed no enhancement of the malformed vessels (Figure 4D). Furthermore, intraoperative cortical evoked potentials remained stable, and the feeding artery, along with the aneurysmal dilation, was completely resected (Figure 4E). Postoperatively, the child recovered without any neurological deficits. Follow-up thoracic spine MRI revealed no definitive lesions (Figures 5A,B), and subsequent complete spinal angiography confirmed the absence of spinal vascular malformations (Figure 5C). The patient's family expressed high satisfaction with the surgical outcome and provided written informed consent for the publication of the imaging data.

## Discussion

SCVMs consist of a heterogeneous group of pathological vascular entities that directly or indirectly affect the spinal cord parenchyma. According to Ling et al. (5), who summarized over 3,000 cases of patients with spinal cord vascular malformations across various age groups, subdural lesions are classified into five subtypes: cavernous vascular malformations, capillary telangiectasias, spinal cord arteriovenous malformations, dural arteriovenous fistulas, and aneurysms. The incidence of each subtype in children significantly differs from that in adults. For instance, spinal dural arteriovenous fistulas (SDAVF), which account for 40% of the total population, are exceedingly rare in children. The etiology of spinal cord vascular malformations remains unclear. However, research by Hong et al. (6) has shown that somatic mutations in the KRAS/BRAF genes are associated with the occurrence of cerebral and spinal vascular malformations. Laberge-le (7), Liquori (8), and Bergametti (9) have suggested that the occurrence of cavernous malformations may be linked to heterozygous functional loss mutations in the CCM1 (KRIT1), CCM2, and CCM3 (PDCD10) genes within families. Hassler et al. (10) believe that spinal dural arteriovenous fistulas are likely acquired, with trauma, infection, and surgery all being potential causes of these lesions. According to Hvingelby et al. (11), mutations in the endoglin gene lead to the development of Capillary Telangiectasia Type I, while mutations in the Activin A Receptor Type II-like 1 gene cause the emergence of Capillary Telangiectasia Type II.

The pathophysiology of spinal cord dysfunction caused by SCVMs is complex and multifaceted, involving mechanisms such as hemorrhage, vascular steal, venous congestion (venous hypertension), and mass effect. These factors may act independently or synergistically, with their relative influence varying at different stages of the disease, thereby contributing to the complexity of the condition. Hemorrhage is the most common manifestation of subdural spinal arteriovenous malformations (12), typically presenting as acute pain in the affected region. This is often accompanied by motor and sensory deficits below the level of the affected spinal segment, along with potential urinary and bowel dysfunction. These symptoms may progressively worsen over several hours or days. In cases where the lesion is located in the upper cervical segments, patients may experience respiratory difficulties or even sudden cardiac arrest. Rupture of perimedullary lesions or aneurysmal structures on feeding arteries or draining veins can result in subarachnoid hemorrhage. Due to the narrow and elongated nature of the spinal subarachnoid space, hemorrhage can extend both upward and downward, potentially reaching the cranial subarachnoid space and ventricles (13). Consequently, evaluation of spinal vascular malformations is critical, particularly in children presenting with subarachnoid hemorrhage, especially if it involves the fourth ventricle. Large or multiple arteriovenous fistulas within the lesion can permit an excessive amount of arterial blood to bypass the capillary network and directly enter the venous system, resulting in inadequate spinal cord perfusion and chronic spinal ischemia. This can manifest as progressive limb muscle atrophy and motor function impairment. Vascular malformations that allow a high flow of arterial blood to enter the venous system directly can elevate venous pressure, leading to spinal edema and subsequent progressive spinal dysfunction. Type III perimedullary arteriovenous fistulas, as well as extradural and paraspinal arteriovenous malformations, are more likely to cause mass effects due to direct compression of the spinal cord, dural sac, or nerve roots by the arteriovenous malformation nidus or expanded venous pouches (14). In this case, the child presented with a clinical manifestation of headache due to subarachnoid hemorrhage and hematoma in the fourth ventricle, while imaging revealed a thoracic spinal vascular malformation.

SCVMs in children are relatively rare and often present with non-specific clinical symptoms, making misdiagnosis common. Accurate diagnosis of SCVMs heavily depends on imaging findings, with spinal DSA being regarded as the “gold standard.” DSA provides a detailed visualization of the feeding arteries, lesion sites, fistulas, and draining veins through super-selective angiography, offering unparalleled precision in diagnosis (15). MRI serves as the primary imaging modality for the early detection and diagnosis of SCVMs. It is a non-invasive, highly effective technique that vividly displays abnormalities such as spinal cord congestion, edema, infarction, and hemorrhage. The high-resolution capabilities of MRI allow clinicians to assess spinal cord injuries and abnormal vasculature more effectively, significantly improving the detection rate of SCVMs. T2-weighted imaging (WI) and enhanced MRI offer clearer visualization of blood vessels, making them the preferred methods for evaluating SCVMs (1). Magnetic resonance angiography (MRA), another vascular imaging technique, is non-invasive, free from radiation, and does not require iodinated contrast agents. However, its resolution is limited compared to DSA, which may hinder precise localization within the lesion. CTA excels in depicting the structural details of SCVMs, especially in cases of spinal dural arteriovenous fistulas. CTA not only detects tortuosity and dilation of perimedullary veins but also has a high accuracy rate in identifying the fistula site. Despite the advantages of rapid scanning and wide coverage (16), most studies have focused on spinal dural arteriovenous fistulas, with less emphasis on other types of SCVMs. Additionally, the radiation exposure associated with CTA remains a concern, particularly in pediatric patients, warranting further exploration of its practical value in diagnosing pediatric SCVMs.

In our case, initial abnormalities were detected through MRI screening, and comprehensive spinal angiography subsequently confirmed the diagnosis of pediatric SCVMs. Treatment options for pediatric SCVMs primarily include microsurgical resection and endovascular embolization. While a few studies have explored the use of radiation therapy in treating adult spinal cord vascular malformations (17), this approach is still relatively immature compared to interventional and microsurgical methods, and should be used with caution in pediatric patients. Endovascular embolization offers distinct advantages, such as being minimally invasive and relatively straightforward. Compared to microsurgical intervention, endovascular treatment helps preserve spinal stability, potentially reducing the risk of future spinal deformities. For children with spinal dural arteriovenous fistulas, microsurgical resection is often preferred, as it allows for precise identification and disconnection of the fistula site and the draining veins. This technique also addresses extradural feeding arteries concurrently, ensuring complete lesion removal, low recurrence rates, and favorable long-term outcomes. It is widely endorsed and recommended by most neurosurgeons (18). Research by Wang et al. (19) supports the view that microsurgical resection is more suitable than endovascular embolization for these cases. In cases of intramedullary spinal arteriovenous malformations in children, the primary treatment strategy involves severing the feeding arteries and occluding the draining veins to reduce venous pressure, improve spinal blood supply, alleviate vascular steal, and restore neurological function. Additionally, removing the malformation and clearing any hematoma helps to alleviate the mass effect. Microsurgical resection is typically indicated for cavernous hemangiomas in children, especially for lesions that have bled or are asymptomatic but accessible on the pial surface. Microsurgical resection remains the only curative treatment for cavernous vascular malformations (11). For perimedullary arteriovenous malformations (PMAVFs) in children, the literature supports the use of embolization, including for Type I PMAVF, with favorable clinical outcomes reported (20, 21). Therefore, embolization should be considered the treatment of choice for pediatric PMAVFs. In our case, given the presence of abnormal signal intensity in the thoracic segment and significant spinal compression evident on MRI, we employed advanced microsurgical techniques, including intraoperative monitoring of evoked potentials and intraoperative fluorescence angiography, to successfully excise the malformed vessels. Postoperatively, the child experienced a positive prognosis.

The distinctiveness of our case lies in the relative rarity of pediatric SCVMs. Initially presenting with a headache indicative of intracranial subarachnoid hemorrhage, it became essential to consider SCVMs as a differential diagnosis, particularly in pediatric cases, after excluding intracranial vascular anomalies. In our case, preoperative magnetic resonance imaging suggested a mass effect caused by the SCVM, prompting us to proceed with the excision of the malformed vessels. This was carried out using expert microsurgical techniques, including intraoperative monitoring of evoked potentials. The surgery proceeded smoothly, resulting in a favorable prognosis for the child. Our case, supported by a review of relevant literature, highlights the critical importance of early diagnosis and timely intervention in the management of pediatric SCVMs. Although the outcome in this instance was positive, it remains a single case, and further validation through larger sample sizes is necessary. Continued research in this area is essential to improve understanding and treatment of these rare pediatric conditions.

## Data Availability

The original contributions presented in the study are included in the article/Supplementary Material, further inquiries can be directed to the corresponding authors.
